# Identification of the GRAS gene family in the * Brassica juncea* genome provides insight into its role in stem swelling in stem mustard

**DOI:** 10.7717/peerj.6682

**Published:** 2019-04-01

**Authors:** Mengyao Li, Bo Sun, Fangjie Xie, Ronggao Gong, Ya Luo, Fen Zhang, Zesheng Yan, Haoru Tang

**Affiliations:** College of Horticulture, Sichuan Agricultural University, Chengdu, China

**Keywords:** GRAS transcription factor, Genome-wide, *Brassica juncea*, Stem swelling, Expression profile

## Abstract

GRAS transcription factors are known to play important roles in plant signal transduction and development. A comprehensive study was conducted to explore the GRAS family in the *Brassica juncea* genome. A total of 88 GRAS genes were identified which were categorized into nine groups according to the phylogenetic analysis. Gene structure analysis showed a high group-specificity, which corroborated the gene grouping results. The chromosome distribution and sequence analysis suggested that gene duplication events are vital for the expansion of GRAS genes in the * B. juncea* genome. The changes in evolution rates and amino acid properties among groups might be responsible for their functional divergence. Interaction networks and *cis*-regulatory elements were analyzed including DELLA and eight interaction proteins (including four GID1, two SLY1, and two PIF3 proteins) that are primarily involved in light and hormone signaling. To understand their regulatory role in growth and development, the expression profiles of BjuGRASs and interaction genes were examined based on transcriptome data and qRT-PCR, and selected genes (*BjuGRAS3*, *5*, *7*, *8*, *10*, *BjuB006276*, *BjuB037910*, and *BjuA021658*) had distinct temporal expression patterns during stem swelling, indicating that they possessed diverse regulatory functions during the developmental process. These results contribute to our understanding on the GRAS gene family and provide the basis for further investigations on the evolution and functional characterization of GRAS genes.

## Introduction

Transcription factors are known as master genes that bind to the promoter of target genes to activate or repress their expression. Investigating plant transcription factors is an important part of functional genomics research that helps us to understand the regulatory network of various biological processes. GRAS is a superfamily of transcription factors unique to plants and was named after the first three members: GAI, RGA and SCR (Bolle, 2004). Typically, GRAS proteins have a variable N-terminus and a highly conserved C-terminus composed of five structural features: LHR I, VHIID, LHR II, PFYRE, and SAW (Pysh et al., 1999). Based on the sequence, structure and phylogenetic relationships, the rice and *Arabidopsis* GRASs are divided into eight groups, including LISCL, PAT1, SCL3, DELLA, SCR, SHR, LS and HAM (Tian et al., 2004). However, as more GRAS genes are identified, the classification of GRASs in different species is slightly altered. For instance, GRAS proteins are divided into at least 13 groups in the tea plant (Wang et al., 2018) and nine groups in the sacred lotus (Wang et al., 2016).

Genome-wide exploration of the GRAS gene family has been conducted in several plants, including *Arabidopsis* (Tian et al., 2004), grapevine (Grimplet et al., 2016), tomato (Huang et al., 2015), and Chinese cabbage (Song et al., 2014), which comprise of 33, 52, 53 and 48 members, respectively. In plants, some members of the GRAS family have been functionally characterized and shown to play diverse functions (Bolle, 2004; Hakoshima, 2018). Plant GRAS transcription factors are involved in the developmental processes, signal transduction pathways, pathogen and stress responses (Hirsch & Oldroyd, 2009; Davière & Achard, 2013; Ma et al., 2010). Members of PAT1 proteins are crucial in the transduction of light signaling pathways. For instance, SCL21 and PAT1 are positive regulators of phytochrome A signal transduction, and SCL13 participates in the phytochrome B pathway (Torres-Galea et al., 2006; Torres-Galea, Hirtreiter & Bolle, 2013). In addition, PAT1 proteins regulate responses to abiotic stress. Overexpression of VaPAT1 enhances cold, drought and salt tolerance in transgenic *Arabidopsis* (Yuan et al., 2016), while OsGRAS23 improves drought tolerance in rice (Xu et al., 2015). Some genes belonging to LS and HAM groups regulate cell differentiation in meristem formation (Schulze et al., 2010). LISCL, SCR, and SHR proteins are involved in the mechanisms that control plant organ development (Gong et al., 2016; Morohashi et al., 2003). Gibberellins, an important plant hormone, regulates many aspects of plant growth and development. In *Arabidopsis*, DELLA proteins function as repressors of the gibberellin signal transduction (Davière & Achard, 2013; Fukazawa et al., 2014). It was found that SCL3 is a positive regulator of DELLA in the root endodermis which integrates and maintains the functions of the GA pathway (Heo et al., 2011). Furthermore, DELLAs play key roles in the mechanism of light signal transduction during growth (Achard et al., 2007).

Mustard crop (*B. juncea*), an annual or biennial herbage species belonging to the genus *Brassica* in the *Cruciferae* family, is widely cultivated in Asia and Europe (Liu, 1996). *B. juncea* (AABB, 2*n* = 36) is an allotetraploid species derived from the hybridization between diploid species *Brassica rapa* (AA, 2*n* = 20) and *Brassica nigra* (BB, 2*n* = 16). Highly differentiated phenotypes are observed in this crop, and its cultivar types are generally classified on the basis of edible organs: leaf mustard, stem mustard, root mustard, oilseed mustard, and seed stalk mustard (Qi, Zhang & Yang, 2007). Stem mustard is a vegetable with an edible swollen stem grown in China (Yang et al., 2018). Besides being consumed as a fresh plant food, pickled products made from the stem mustard are widely consumed around the world. The swelling of the stem mustard is a complex process and is the most important factor that determines the yield. Temperature, photoperiod, cell division, and endoreduplication are known to influence stem swelling (Guo et al., 1994; Shi et al., 2012). So far, research on stem mustard has been mainly focused on genetics and breeding, physiological, biochemical, and traditional classification. Hence, there are few reports on the molecular mechanism of stem swelling.

Due to its role in plant growth and development processes, GRAS transcription factors have been extensively studied in several plants and applied in various breeding and genetic engineering programs. The whole genome sequence of *B. juncea* has already been unraveled (Yang et al., 2016), which provides a platform for the exploration of the structure, evolution, and biological function of the GRAS family. On this basis, we performed a genome-wide analysis of the GRAS family in *B. juncea* with a focus on gene structures, evolutionary analysis, and expression profiling during the stem swelling period. The results from this study provide a better understanding of the function and mechanisms of the GRAS family in *B. juncea*.

## Materials & Methods

### Identification of GRAS transcription factors in *B. juncea*

All nucleotide and protein sequences were downloaded from the latest version (V1.5) of *B. juncea* genome (http://brassicadb.org/brad/) to build a local database. HMMER3.0 software (http://www.ebi.ac.uk/Tools/hmmer/) was used to identify the GRAS proteins based on the HMM profile (GRAS, PF00011) at default parameters. All GRAS proteins obtained were further searched on the NCBI and Pfam databases to determine the GRAS domain. Sequences lacking the complete GRAS domain were eliminated. The Sequence Manipulation Suite (http://www.bioinformatics.org/sms/) and ExPASy (https://web.expasy.org/) software were utilized to analyze the composition and physical/chemical properties of GRAS proteins (Gasteiger et al., 2003). The position information of all GRAS genes was derived from the *B. juncea* genome database, and the chromosome location was drawn using MapInspect software (https://mapinspect.software.informer.com/).

### Phylogenetic analysis of GRAS transcription factors

The GRAS sequences of *Arabidopsis* were extracted from TAIR (https://www.arabidopsis.org/) (Table S1). The GRAS family in other species were downloaded from the Plant Transcription Factor Database (http://planttfdb.cbi.pku.edu.cn/). Multiple alignments of GRAS protein sequences from *Arabidopsis* and *B. juncea* were performed using ClustalX in default parameters. A phylogenetic tree was generated using MEGA5.0 by the Neighbor-joining method with the 1,000-times bootstrap test.

### Analysis of gene structure, conserved motif, and promoter region

Exon-intron structures of BjuGRASs were examined using the Gene Structure Display Server tool (http://gsds.cbi.pku.edu.cn/). Conserved motifs were predicted by the Conserved Domain Database (https://www.ncbi.nlm.nih.gov/cdd) and MEME Suite (http://meme-suite.org/). The parameters in MEME were set as default value except for the option “MEME should find” which was set to 15, and the motif structure schematic diagrams were drawn using the TBtools software (Chen et al., 2018). The upstream 1,500 bp regions of DELLA genes were downloaded from the genome database. PlantCARE (http://bioinformatics.psb.ugent.be/webtools/plantcare/html/) was used to determine the *cis*-element on gene promoter region (Rombauts et al., 1999).

### Functional divergence analysis

DIVERGE3.0 (Gu et al., 2013) was used to estimate the functional divergence between gene clusters of the GRAS family. The values of type-I and type-II were tested. Type-I represented the functional divergence at the site-specific shift of evolutionary rate, while type-II reflected the changes at the site-specific shift of amino acid physiochemical property.

### Analysis of functional annotation and interaction network

All BjuGRAS proteins were compared using the Gene Ontology (GO) database to investigate their putative functions. Blast2GO (Conesa et al., 2005) was used to obtain the relevant GO ID, and the results of the analysis contained three parts: molecular function, cellular component, and biological process. Specific protein interactions among the GRAS transcription factors in *B. juncea* were constructed using the STRING software (Franceschini et al., 2013).

### Expression analysis of BjuGRASs in stem swelling in stem mustard

To identify the BjuGRAS genes involved in stem swelling in stem mustard, the RNA-seq data generated from three biological replicates of four stem swelling stages was analyzed as follows: stage 1 (stem diameter was 2 cm), stage 2 (stem diameter was 4 cm), stage 3 (stem diameter was 6 cm), and stage 4 (stem diameter was 8 cm). The stem mustard variety of ‘Fuza No.2’ was used for RNA sequencing on the Illumina HiSeq 4000 platform, and the data were deposited on the NCBI website with the accession number SRP151320. The expression abundance of BjuGRAS genes was represented as FPKM (Trapnell et al., 2010). FPKM values were normalized to the mean of the whole data and then transformed by log2. The heatmap was generated by MultiExperiment Viewer software (Saeed et al., 2003).

### RNA extraction and quantitative real-time PCR (qRT-PCR) analysis

Total RNA was extracted from the stem samples at four stem swelling periods (stem diameters were 2 cm, 4 cm, 6 cm, and 8 cm) using the total RNA kit (Tiangen, Beijing, China). For cDNA synthesis, 1.0 µg RNA was reverse transcribed with oligo (dT) and random primers using the Goldenstar™ RT6 cDNA Synthesis Mix (TsingKe, Beijing, China). qRT-PCR was conducted with a Bio-Rad CFX96™ real-time PCR System (Bio-Rad, CA, USA) using 2 ×T5 Fast qPCR Mix (SYBRGreenI) (TsingKe, Beijing, China). All primers used are shown in Table S2. All experiments were performed with three independent RNA samples, and *Actin2* (*BjuB008540*) was used as the internal control to normalize the gene expression. The relative mRNA expression was calculated using the 2^−ΔΔ*CT*^ method (Pfaffl, 2001).

## Results

### Identification of GRAS transcription factor family in *B. juncea*

After screening on the *B. juncea* genome database, a total of 88 genes encoding GRAS proteins were identified and renamed as BjuGRAS1∼BjuGRAS88. The gene nomenclature, length of amino acid, protein molecular weight, theoretical isoelectric point (*pI*), grand average of hydropathicity (GRAVY), and amino acid composition are detailed in Table S3. The deduced length of GRAS proteins ranged from 318 to 792 amino acids, the molecular weights ranged from 35.79 kD to 88.74 kD, and the *pI* ranged from 4.66 to 8.45. In terms of the amino acid composition, aliphatic amino acids accounted for the largest proportion with an average of 20%, while aromatic amino acids were only 8%. The grand average of hydropathy (GRAVY) values of all GRAS proteins were less than zero, suggesting that these proteins were hydrophilic.

### Phylogenetic relationship and distribution of GRAS family factors

**Figure 1:**
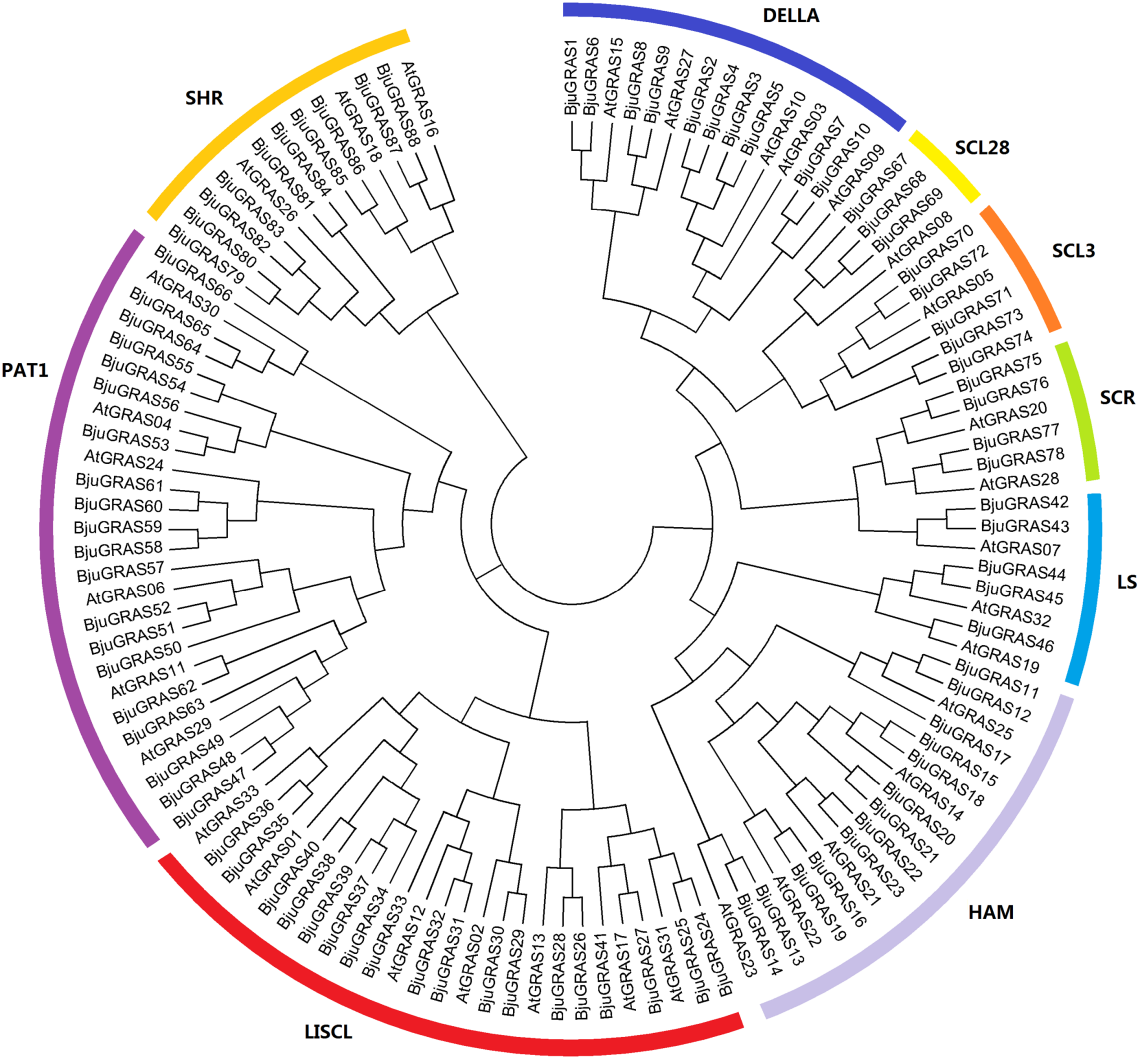
Phylogenetic tree of GRAS proteins from * Brassica juncea* and *Arabidopsis*. The phylogenetic tree was constructed by the Neighbor-joining method with 1,000 bootstrap replications. The nine groups are represented with different colors.

To understand the phylogenetic relationship and classification of GRAS genes, a phylogenetic tree was constructed based on multiple sequence alignment of 88 proteins from *B. juncea* and 33 from *Arabidopsis*. All GRAS proteins were divided into nine cluster groups: LISCL, PAT1, SCL3, DELLA, SCR, SHR, LS, SCL28 and HAM (Fig. 1). This classification was slightly different from other plants, such as tea plant (Wang et al., 2018) and sacred lotus (Wang et al., 2016). The PAT1 and LISCL groups were the two largest subfamilies which accounted for 21% and 22% of the total GRAS genes, respectively, while SCL28 was the smallest group having three members in *B. juncea* and one member in *Arabidopsis*.

The GRAS family factors have been comprehensively identified in many species based on whole-genome sequencing. To compare the distribution of the GRAS family among species, we analyzed the number of GRASs in 20 species, ranging from algae to higher plants. As summarized in Table 1, the GRAS genes were exclusively found in higher plants, and no member was identified in lower plants (*Klebsormidium flaccidum*, *Volvox carteri*, *Ostreococcus lucimarinus*). This indicated that the GRAS genes evolved from lower plants to higher plants. The number of GRAS families varied greatly among different species in higher plants. Among them, *Brassica napus* contained 92 members, followed by *B. juncea* (88) and *Zea mays* (86). *Marchantia polymorpha* had the lowest number with 11 GRASs according to our analysis. All groups were widely present in many species except for *Marchantia polymorpha* which did not have the PAT1 and SCL3 groups, and *Brassica oleracea* which had no SCL28 gene. SCL9 was the largest group, followed by PAT1 and HAM. There were few SCL28 genes compared to other groups.

**Table 1:** Comparison of GRAS transcription factors among different species.

Taxonomic Group	Species	Classification									Total
		PAT1	SCL9	SHR	SCR	LAS	HAM	SCL28	SCL3	DELLA	
Dicotyledon	*Arabidopsis thaliana*	6	7	3	2	3	5	1	1	5	33
	*Brassica rapa*	12	10	5	2	2	8	2	2	5	48
	*Brassica juncea*	20	18	10	4	4	14	3	5	10	88
	*Brassica oleracea*	7	8	5	1	2	6	0	2	4	35
	*Brassica napus*	23	19	7	4	4	16	4	5	10	92
	*Nelumbo nucifera*	9	2	7	2	5	5	1	2	5	38
	*Medicago truncatula*	11	21	9	3	1	7	2	11	2	67
	*Solanum tuberosum*	17	21	6	3	5	6	4	3	5	70
Monocotyledon	*Zea mays*	10	24	9	4	4	15	5	8	7	86
	*Oryza sativa*	8	15	5	4	5	8	3	8	4	60
	*Sorghum bicolor*	7	39	7	4	3	8	2	6	4	80
	*Phyllostachys heterocycla*	13	13	4	3	1	13	2	5	5	59
	*Setaria italica*	10	18	7	4	3	8	1	6	5	62
Basal Magnoliophyta	*Amborella trichopoda*	3	11	5	4	2	7	1	3	7	43
Coniferophyta	*Picea abies*	12	1	2	3	2	6	1	1	1	29
Marchantiophyta	*Marchantia polymorpha*	0	1	2	1	1	1	1	0	4	11
Bryophyta	*Physcomitrella patens*	2	7	9	5	6	5	2	5	2	43
Charophyta	*Klebsormidium flaccidum*	0	0	0	0	0	0	0	0	0	0
Chlorophyta	Volvox carteri	0	0	0	0	0	0	0	0	0	0
	*Ostreococcus lucimarinus*	0	0	0	0	0	0	0	0	0	0

### Gene structure analysis of GRAS genes

**Figure 2:**
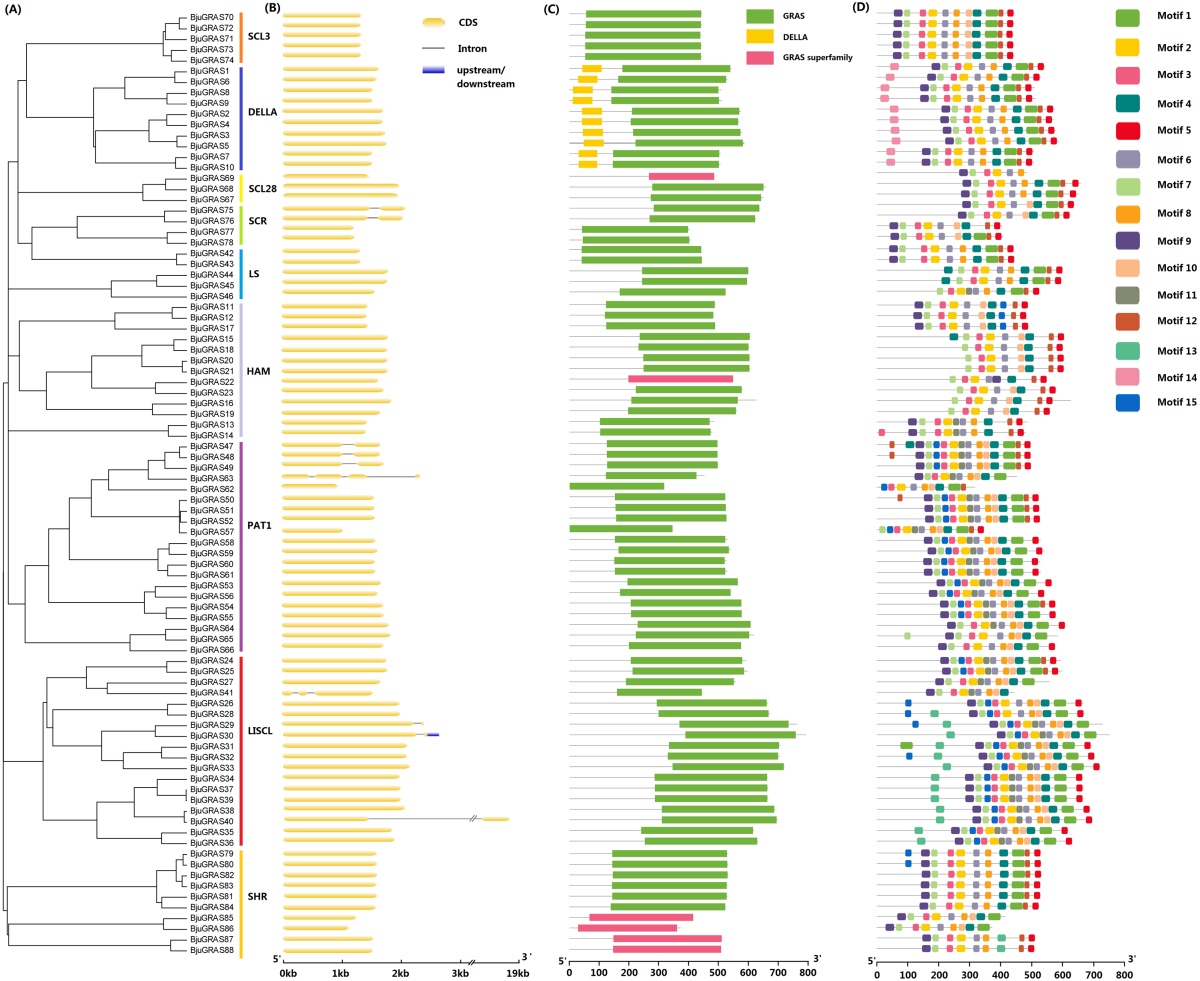
Structure analysis of BjuGRAS genes. (A) Phylogenetic tree of GRAS proteins in * Brassica juncea*. (B) Exon-intron structure of BjuGRAS genes. (C) The conserved domains in BjuGRAS proteins. (D) Distribution of motifs of BjuGRAS proteins.

Gene structure among GRAS family groups was compared in order to analyze their differences. Only ten BjuGRAS genes had one or more introns: two, four, and four for SCR, PAT1, and LISCL groups, respectively (Figs. 2A–2B). The remaining 89% of the BjuGRAS genes did not contain any intron, which is similar to the exon-intron structural characteristic of this family in other species. For example, 88%, 83.3%, and 80.23% of GRAS genes in grapevine, Chinese cabbage, and maize had only one exon, respectively (Song et al., 2014; Guo et al., 2017).

The conserved motifs among sequences were predicted to provide insights into the structural relationships. It was discovered that the C-terminus of GRAS proteins is highly conserved in terms of sequence homology (Figs. 2C–2D). The GRAS domain was identified in all BjuGRASs, and the DELLA structure was only found in the DELLA group (Fig. 2C). Fifteen distinct motifs were identified and used to distinguish each group (Fig. 2D and Table 2). Members of each group were highly similar in terms of motif composition but differed from members of other groups. The common motif in all BjuGRASs was motif 2 which contained a VHIID sequence. Several motifs were found in one or two groups; for example, motif 14 in DELLA, motif 13 in LISCL, motif 11 in PAT1, and motif 15 in LISCL. High sequence similarities in BjuGRASs also supported the phylogenetic relationship and the classification of group clusters.

### Chromosomal distribution and duplication of BjuGRAS genes

**Figure 3:**
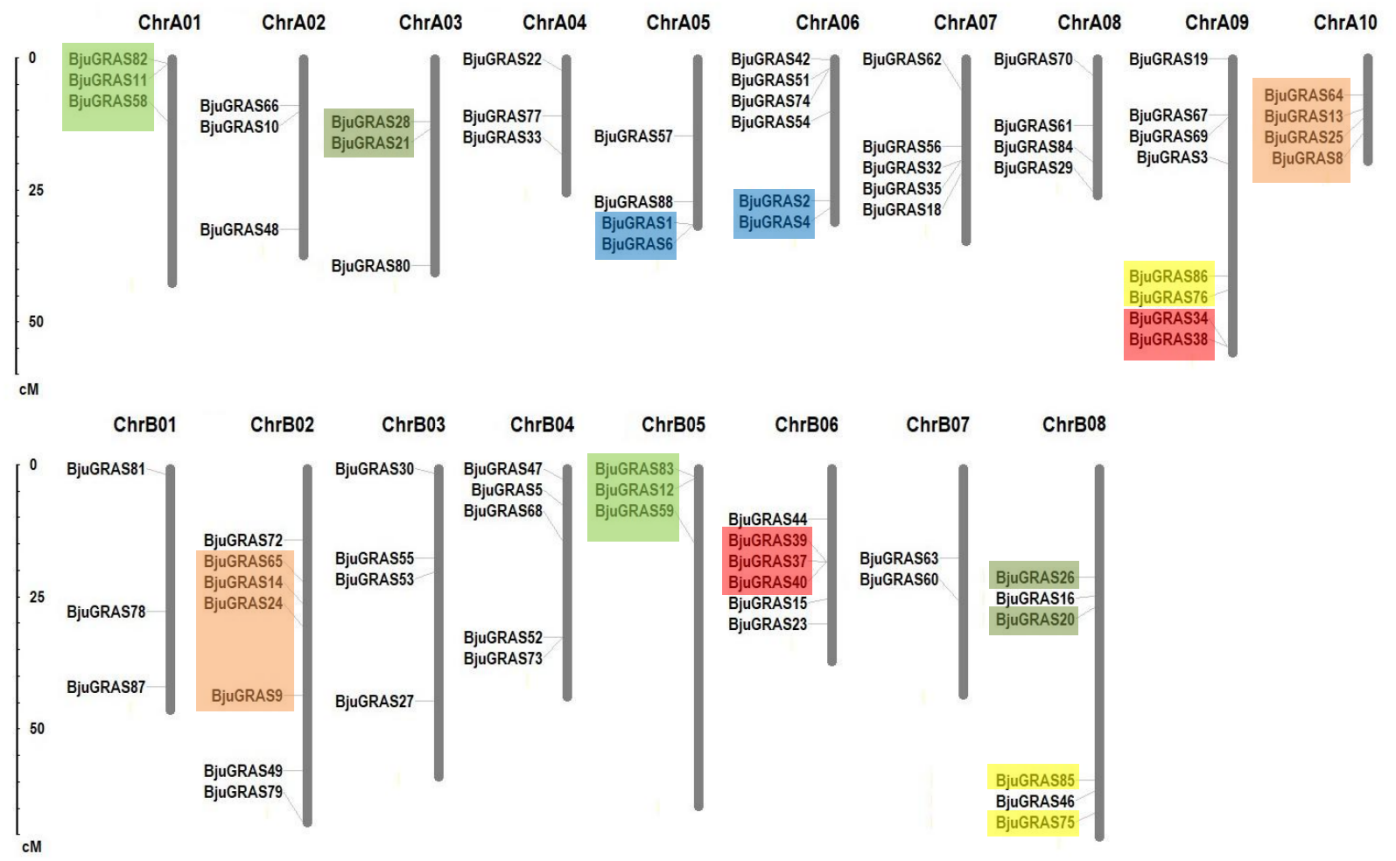
Chromosomal distribution and gene duplications of GRAS genes in the *Brassica juncea* genome. The segmental duplicated are represented with different colors. The scale bar on the left indicates the size of chromosomes.

Except for the nine genes that were located on unanchored contigs or scaffolds, the remaining 79 BjuGRAS genes were unevenly distributed in all 18 chromosomes (Fig. 3). Among them, 43 genes were found on the A subgenome and 36 genes on the B subgenome. ChrA09 harbored the highest number of BjuGRAS genes (eight genes), followed by seven genes on ChrB02, and six genes on ChrA06, ChrB06, and ChrB08. Gene duplication events, including tandem duplication, dispersed duplication, and chromosomal segmental duplications were also found in the chromosomes. According to the localization and sequence alignment analysis of BjuGRAS genes, at least 38 gene pairs with more than 90% sequence similarity were found to be homologous genes, 30 pairs of which were a one-to-one match between the A subgenome and B subgenome (Table S4). Several hotspots of GRAS genes clusters were observed among the chromosomes. For example, *BjuGRAS37*, *BjuGRAS39*, and *BjuGRAS40* were clustered on a 15-kb segment in ChrB06 whereas *BjuGRAS32*/*BjuGRAS35* and *BjuGRAS34*/*BjuGRAS38* were clustered on ChrA07 and ChrA09 with a distance of less than 5 kb, respectively. In addition, multiple gene pairs were clustered in some regions, such as large sections of ChrA01, ChrB05, ChrA10, and ChrB02, which may be attributed to chromosomal segmental duplications.

**Table 2:** Motif sequences identified by MEME tools.

Motif	Length of amino acid	Best possible match
Motif 1	41	VVEZECLGREIVNVVACEGSERVERHETLGKWRVRMMMAGF
Motif 2	29	VHIIDFDIGQGFQWPSLIQALASRPGGPP
Motif 3	21	FYEVCPYLKFGYFTANQAILE
Motif 4	29	VEQEGNHNTPPFLTRFKEALHYYSALFDS
Motif 5	21	DDGWLLLGWKDRPLVTVSAWK
Motif 6	21	TGERLAQFAKSLGVPFEFNAV
Motif 7	21	PSGDPMQRLAAYFAEALAARJ
Motif 8	21	PGEALAVNSVFRLHNLPDESV
Motif 9	29	DLRSLLLECAQAVSSGDLALANALLKZJR
Motif 10	21	VENPRDRLLRLIKSLSPKVVT
Motif 11	21	KLRITGIDDPQSGFRPGGGLE
Motif 12	17	KPVPLSSYAAKQAKLLL
Motif 13	28	SMFSDAESAMQFKRGLEEASKFLPNSDQ
Motif 14	29	DELLAVLGYKVRSSEMAEVAQKLEQLETV
Motif 15	21	SSIYKALKSKKPTAAEILKAY

### Estimation of functional divergence

Due to the number limits (at least four sequences in clusters) used in the DIVERGE3.0 tool, LS and SCL28 groups were excluded during functional divergence analysis. Pairwise comparisons of protein sequences among the seven groups were performed to estimate the values of divergence coefficient for type I and type II, and the results are presented in Table 3. The difference in the evolution rate of GRAS genes after gene replication is reflected by the significant variability of the specific amino acid site. The greater the divergence coefficient, the greater the variability. The divergence coefficient for type I (*θ*_I_) was higher than 0 and ranged from 0.2072 to 0.9992, which suggested that there was a significant difference in the functional divergence among GRAS groups. The divergence coefficient for type II (*θ*_II_) was also found, indicating that the physical and chemical properties of amino acids were significantly changed. Collectively, these results suggested that GRAS groups may have undergone varying degrees of functional divergence after gene duplication. Moreover, a posteriori probability (Qk) was used to show that divergence-related residues were responsible for functional divergence. Qk >0.95 was used as the cut-off for defining the critical amino acid sites (CAASs) between GRAS groups. Seven paired groups containing 358 CAASs were detected in type I analysis, of which 115, 115, and 114 were identified in SCL3/LISCL, SCL3/PAT1, and DELLA/LISCL, respectively. A total of 536 CAASs were identified in type II, of which the most highly represented paired groups were DELLA/SCL3, SCL3/HAM, and SCL3/SCR. Compared to type I, many CAASs were identified in more pairs in type II, suggesting that the functional divergence among GRAS genes might be attributed to the change in amino acid property, followed by site-specific shifts in evolutionary rate.

**Table 3:** Functional divergence analysis of different groups of GRAS family.

	Type-I				Type-II		
	*θ*_I_	SE	LRT	Qk>0.95	*θ*_II_	SE	Qk>0.95
DELLA/SCL3	0.7344	0.3514	4.3690	0	0.4356	0.0866	57
DELLA/SCR	0.2072	0.1358	2.3290	0	0.2753	0.1065	44
DELLA/HAM	0.3272	0.1931	2.8698	0	−0.0007	0.2869	42
DELLA/PAT1	0.7088	0.1877	14.2671	0	0.0117	0.2700	21
DELLA/LISCL	0.9848	0.1992	24.4302	114	0.2327	0.1922	48
DELLA/SHR	0.4112	0.2609	2.4841	0	0.0582	0.2460	35
SCL3/SCR	0.3720	0.2581	2.0781	0	0.3889	0.0898	53
SCL3/HAM	0.3824	0.6102	0.3927	0	−0.0081	0.3046	56
SCL3/PAT1	0.9856	0.5617	3.0789	115	−0.0600	0.2769	13
SCL3/LISCL	0.9992	0.3529	8.0169	115	0.1925	0.1944	39
SCL3/SHR	0.5120	0.8816	0.3373	0	−0.0944	0.2741	0
SCR/HAM	0.1792	0.1557	1.3249	0	−0.2762	0.3196	0
SCR/PAT1	0.4912	0.1580	9.6638	0	−0.1285	0.2827	6
SCR/LISCL	0.3905	0.1630	5.7409	0	0.0361	0.2087	42
SCR/SHR	0.4224	0.2029	4.3347	0	0.0095	0.2481	39
HAM/PAT1	0.6208	0.1308	22.5304	3	−0.3747	0.5464	0
HAM/LISCL	0.7728	0.1393	30.7679	6	−0.1956	0.4379	0
HAM/SHR	0.3280	0.1593	4.2390	0	−0.6509	0.6047	0
PAT1/LISCL	0.5923	0.1304	20.6357	1	−0.3215	0.4229	0
PAT1/SHR	0.5208	0.1435	13.1721	0	−0.6904	0.5410	0
LISCL/SHR	0.8632	0.1971	19.1818	4	0.1313	0.3453	41

### Functional annotation of BjuGRAS genes

**Figure 4:**
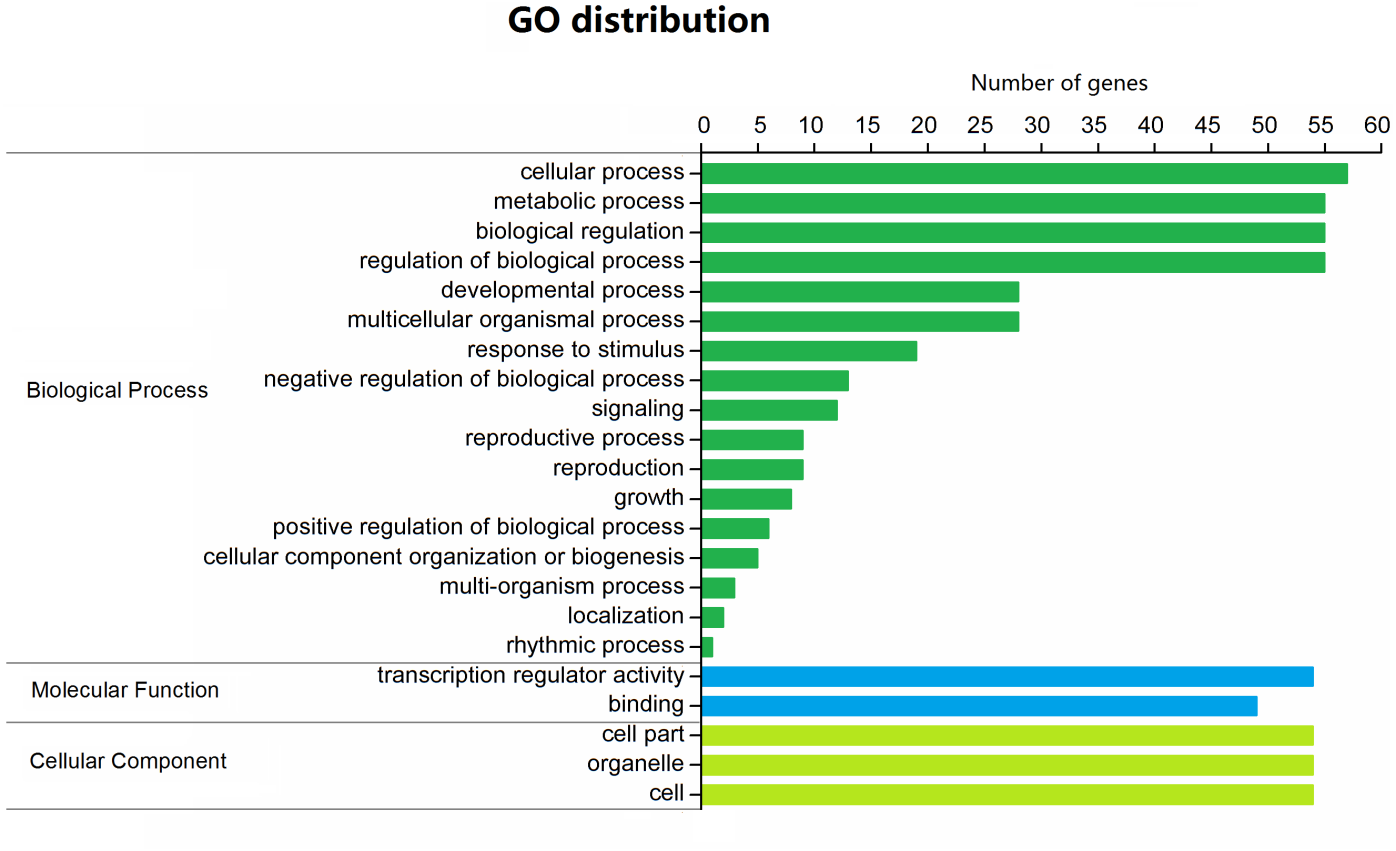
GO function classification of BjuGRAS genes. The annotation results were classified into three GO categories on the basis of GO second level terms.

All 88 BjuGRAS genes were analyzed by Blast2GO to investigate their functional annotation. A total of 59 members were mapped on the GO database, resulting in 412 annotations (Table S5). The annotation results were classified into 22 function terms, which were in turn assigned into three ontology categories: biological process (BP), cellular component (CC), and molecular function (MF). The GO categories were further summarized on the basis of GO second level terms (Fig. 4). Within BP, the top four groups were ‘cellular process’, ‘metabolic process’, ‘biological regulation’ and ‘regulation of biological process’. Under the CC category, three terms were identified: ‘cell part’, ‘organelle’, and ‘cell’. In the MF category, 54 genes were related to ‘transcription regulator activity’ and 49 were related to ‘binding’.

### Interaction network analysis of BjuGRAS proteins

**Figure 5:**
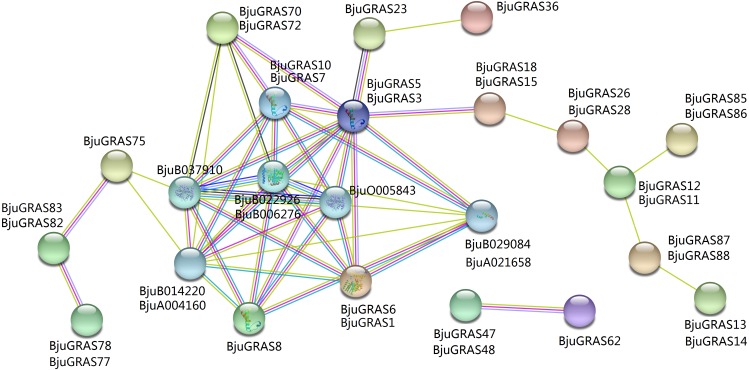
Interaction network of BjuGRAS factors in * Brassica juncea* according to orthologs in * Arabidopsis*.

To explore the regulatory relationship of GRAS transcription factors in *B. juncea*, an interaction network was constructed according to the orthologs in *Arabidopsis*. A total of 31 GRAS proteins and eight proteins in *B. juncea* were found in the complex interaction network map (Fig. 5). Seven proteins (BjuGRAS1, 3, 5, 6, 7, 8, 10), which belong to the DELLA group, showed significant correlations with several other proteins. Except for these GRAS proteins, the rest were homologous to SLY1 protein (BjuA004160, BjuB014220), PIF3 protein (BjuB029084, BjuA021658), and GID1 family protein (BjuB006276, BjuB022926, BjuB037910, BjuO005843). These relationships provide the first glimpse of the functions of BjuGRAS genes in the regulatory mechanisms of biological functions.

### Promoter region analysis of DELLA group transcription factors related to light and hormone signaling

**Figure 6:**
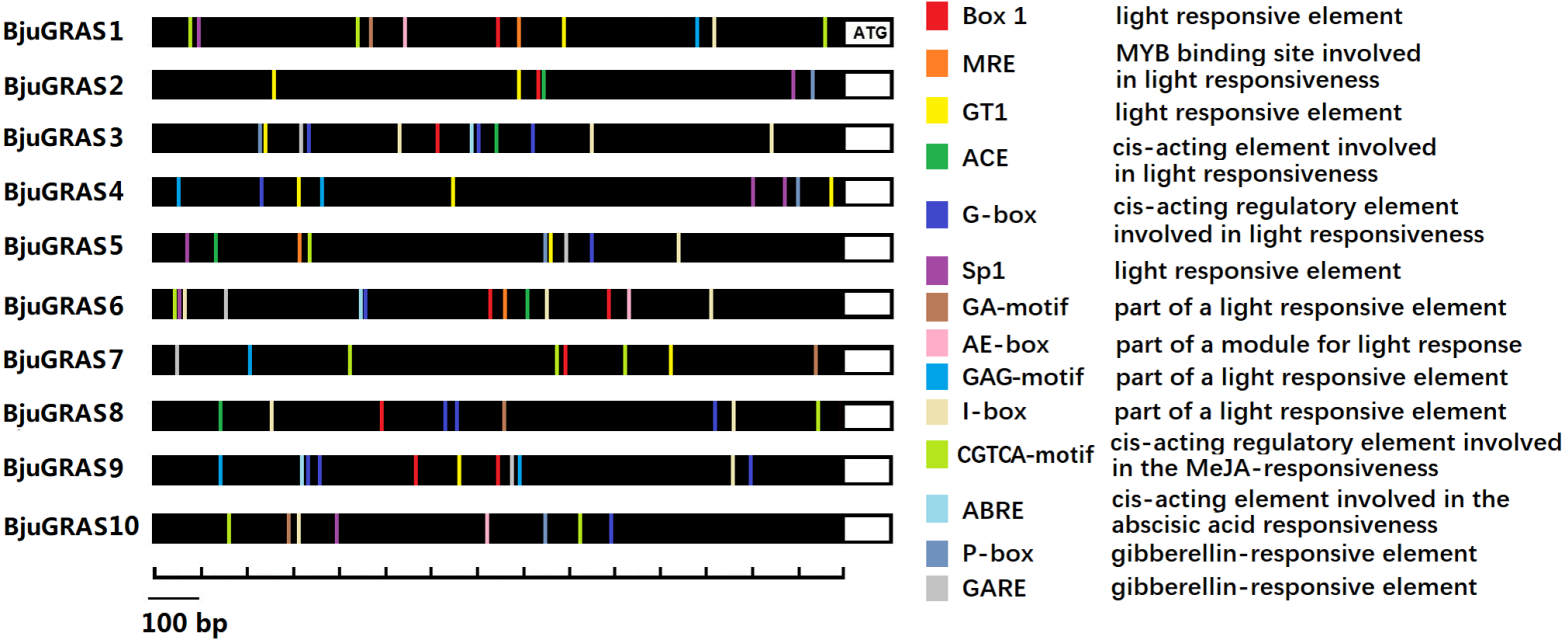
*cis*-regulatory element anlysis of DELLA group members. Different colors indicate different *cis* elements existing in the promoter region.

Prediction and identification of transcription factor binding sites can accelerate the understanding of the mechanisms of transcriptional regulation. Numerous *cis*-elements were identified at the promoter regions of DELLA genes, and at least ten kinds of elements related to light responsiveness and four kinds related to hormone responsiveness were identified in several genes (Fig. 6). Our results showed that the light responsive elements G-box, I-box, GT1, and Box 1 appeared 13, 12, 10, and nine times, respectively, while gibberellin responsive elements P-box and GRAE appeared five times each. The MeJA-responsive element (CGTCA-motif) appeared 11 times, of which *BjuGRAS1* and *BjuGRAS7* appeared three times, suggesting that these two genes may be involved in the regulation of methyl jasmonate. *BjuGRAS1*, *3*, and *6* contained more regulatory elements in their promoter regions, which contributed to their high interaction and involvement in regulatory processes.

### Expression analysis of BjuGRAS genes during stem swelling stages

**Figure 7:**
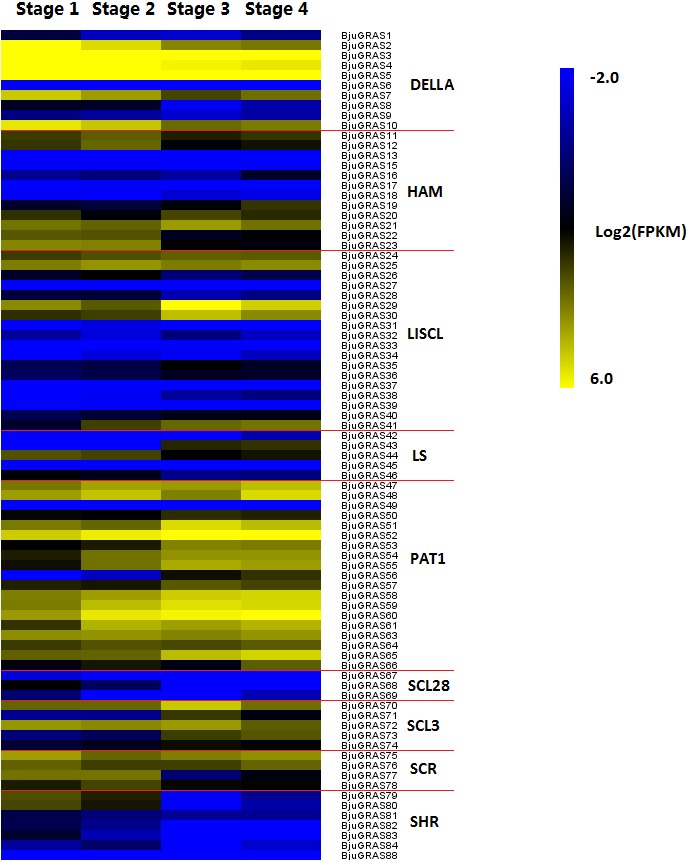
Expression levels of BjuGRAS genes at different developmental stages in stem swelling. Fragments per kilobase per million (FPKM) values of BjuGRAS genes were transformed by log2, and the heatmap was constructed with MultiExperiment Viewer sofware.

The RNA-seq data on the four stages of stem swelling was used to investigate the expression profiles of BjuGRAS genes. The transcript levels of 83 BjuGRAS genes were obtained from at least one stage and five BjuGRAS genes were not detected. It should be noted that the expression difference in stage 1/stage 2 and stage 3/stage 4 comparisons were not significant (Fig. 7). Most genes displayed huge changes from stage 2 to stage 3, which suggested that this stage is important during stem swelling. Most BjuGRAS genes belonging to the same group had a similar expression pattern. For example, genes from PAT1 and SCR displayed a relatively high level in all four stages, while the expression levels of SCL28 and SHR members were relatively low. Analysis of the expression patterns of DELLA genes showed that they were all decreased across the stem swelling stages, but six of them (*BjuGRAS2*, *3*, *4*, *5*, *7*, and *10*) were highly expressed. Among 18 genes encoding LISCL proteins, five were increased and highly expressed during stem development, whereas the expressions of other genes were variable. The diversity of expression patterns among different groups indicated that BjuGRAS genes have special functions at different developmental stages.

### Characterization and expression analysis of interaction genes of DELLA genes during stem swelling stages

**Figure 8:**
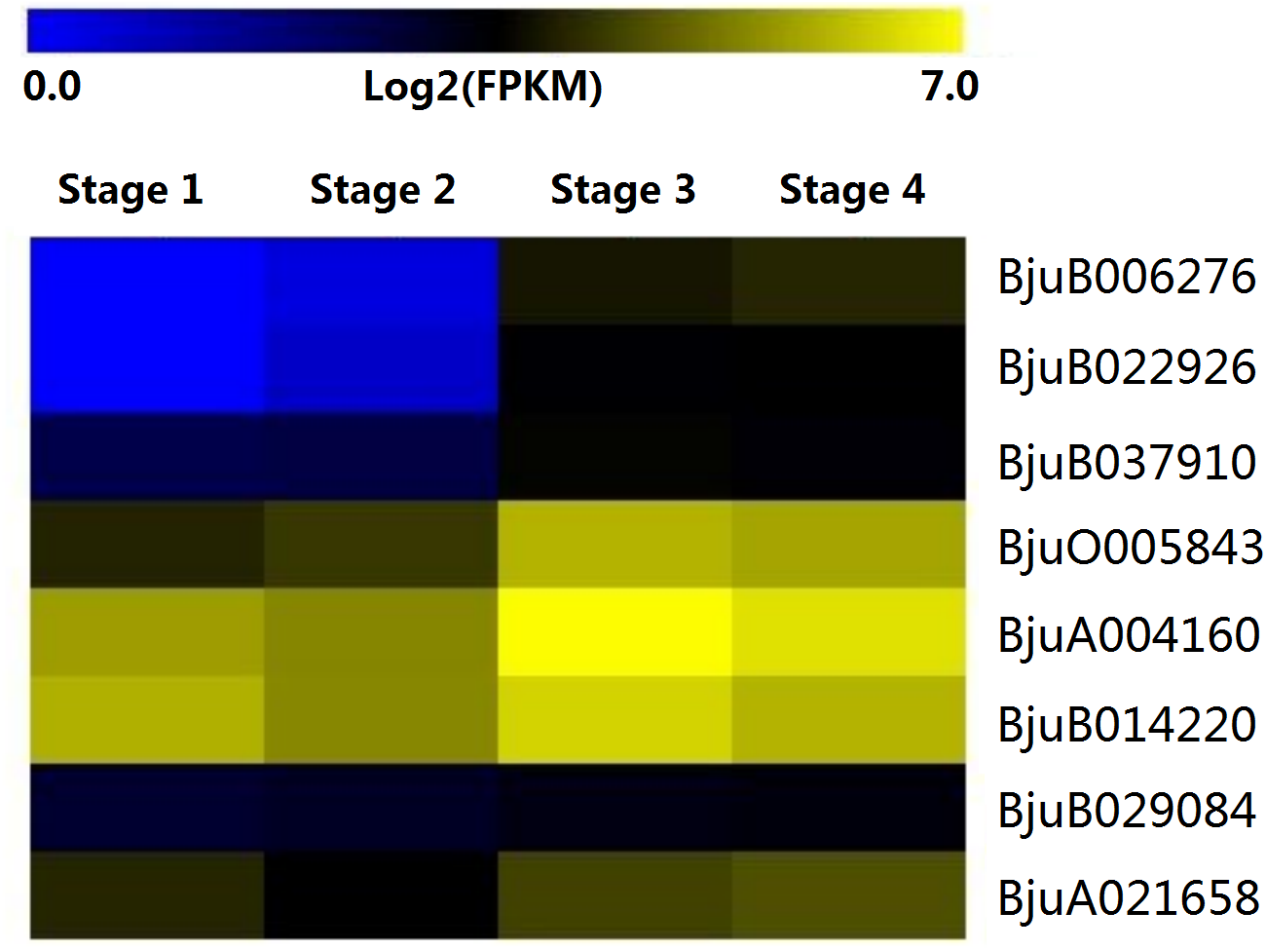
Expression levels of interaction genes of DELLA genes at different developmental stages in stem swelling.

The expression patterns of eight genes identified from interaction analysis were calculated over the entire course of stem development (Fig. 8). The expression pattern of four GID1 genes (*BjuB006276*, *BjuB022926*, *BjuB037910*, *BjuO005843*) was opposite to that of DELLA genes. Their expression was up-regulated during stem swelling, especially from stage 2 to stage 3. PIF3 genes showed a more stable expression throughout the four stages. However, *BjuB029084* was lowly expressed, while *BjuA021658* displayed a relatively high expression level. Two SLY1 genes, *BjuA004160* and *BjuB014220*, maintained a high expression level throughout stem development. These results demonstrated that the genes recognized in previous interaction analyses might be involved in the regulation of stem swelling.

**Figure 9:**
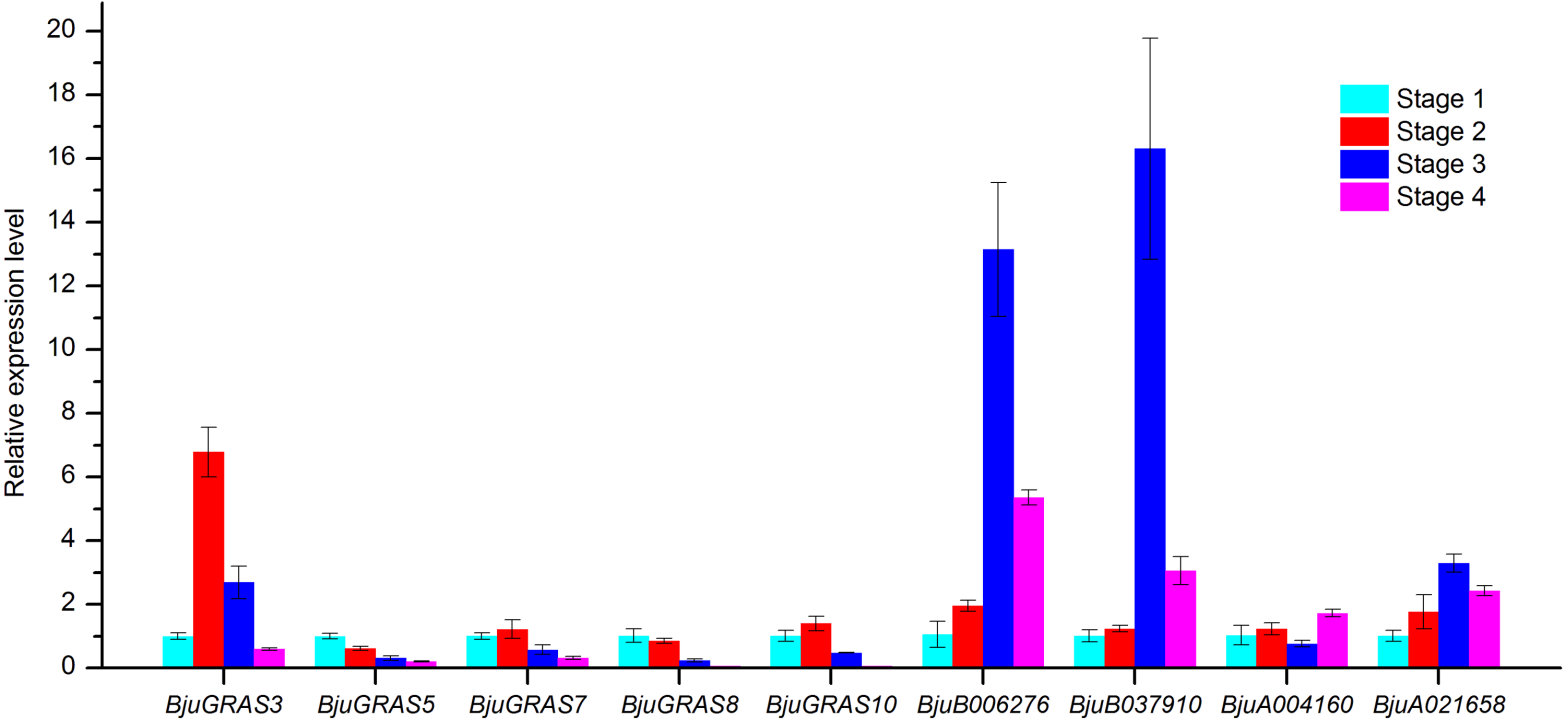
Analysis of gene expressions during four stem swelling stages by qRT-PCR.

### Analysis of genes involved in stem swelling by qRT- PCR

To validate whether *DELLA* genes and interaction genes were expressed in the stem and whether they were involved in stem swelling, nine genes were analyzed using qRT-PCR. The result showed that the nine genes were differentially expressed during stem swelling stages (Fig. 9). The expression level of *BjuGRAS3* had a seven-fold increase in stage 2, while its expression in stage 3 and stage 4 was down-regulated. The remaining four DELLA genes (*BjuGRAS5*, *7*, *8*, *10*) were remarkably down-regulated during the stem swelling process and were lowly expressed in the later stages of stem swelling as determined by RNA-seq (Fig. 7). Two GID1 genes (*BjuB006276* and *BjuB037910*) were remarkably up-regulated from stage 2 to stage 3, and with higher expression level in stage 3 and stage 4. *BjuA021658* expression was increased during the four stages, but the expression of *BjuA004160* was variable. This finding further demonstrated that the selected genes were extensively involved in stem swelling.

## Discussion

### Identification of GRAS transcription factors in *B. juncea*

*Brassica* is an important genus of the *Cruciferae* family and contains many crops with economic value. *B. rapa* (AA, 2*n* = 20), *B. oleracea* (CC, 2*n* = 18), *B. nigra* (BB, 2*n* = 16), *B. napus* (AACC, 2*n* = 38), *B. juncea* (AABB, 2*n* = 36), and *Brassica carinata* (BBCC, 2*n* = 34) were the most representative species, and the genetic relationship among these six *Brassica* species are called the ‘U-triangle’ model (Nagaharu, 1935). In the ‘U-triangle’ model, there are three basic subgenomes (A subgenome, B subgenome, and C subgenome). The mustard crop is an economically and nutritionally important vegetable. Genome sequencing for *Brassica* provides nearly all information needed to explore the evolution and functional diversity of genes. As an allotetraploid plant, *B. juncea* originated from the natural hybridization between *B. rapa* and *B. nigr* a. According to the ‘U-triangle’ theory, the ratio between the number of genes in *B. juncea* and *B. rapa* is about 2:1. The present study identified 88 GRAS genes in the *B. juncea* genome, which is about two times the number of GRAS genes in *B. rapa* and three times the number of GRAS genes in *Arabidopsis*. For many other gene families, several studies revealed that gene duplication is the main reason for gene family expansion (Nakano et al., 2006; Kong et al., 2007). Homologous gene pairs and gene clusters were found on the A subgenome and B subgenome, suggesting that tandem and segmental duplication events have occurred in genomic rearrangements and expansions.

The structure of the phylogenetic tree of GRAS genes in *B. juncea* and *Arabidopsis* indicated that GRAS genes can be classified into nine groups. Structural analyses provided more information needed to understand the evolution patterns and functional divergence. The shift in amino acid site-specific patterns may contribute to functional divergence during evolution (Lichtarge, Bourne & Cohen, 1996; Gu, 1999; Sun et al., 2002). Functional divergence analysis of type I and II coefficients showed that GRAS genes in all groups were functionally divergent from each other, indicating that change had occurred in evolutionary rates and amino acid property. Similarities in motif composition were observed in the same group, which may have contributed to the diverse functions of the GRAS family. In addition, the highly conserved motifs of BjuGRASs supported the phylogenetic relationship and classification of group clusters.

### The role of BjuGRAS genes in stem swelling

GRAS proteins are a family of plant-specific transcription factors that play pivotal roles in plant growth and development (Bolle, 2004; Hirsch & Oldroyd, 2009). The swelling stem is the main edible organ of the stem mustard. The process of stem swelling is complex in terms of physiological and biochemical mechanisms as it involves a series of regulatory networks such as morphogenesis, nutrient accumulation, and gene expression. Exploring the differential expression of genes may reveal the expansion mechanism of the stem at the molecular level, and also provide a theoretical basis for genetic improvement. GRAS genes in several plant species, especially in the model plant *Arabidopsis*, have been demonstrated to express in a spatial and temporal manner during plant development (Tian et al., 2004; Lee et al., 2008; Yoon et al., 2016). Even so, little is known about their function in the development of *B. juncea*. Here, expression patterns of all BjuGRAS genes were examined in different developmental stages. These results revealed that GRAS genes of the same group displayed similar expression pattern at specific stages, but significant differences were observed among groups, reflecting their involvement in diverse developmental processes. Moreover, qRT-PCR analysis revealed that the expression patterns of five *DELLA* genes (*BjuGRAS3*, *5*, *7*, *8*, *10*) were consistent with the RNA-seq data, which further supported a role for these genes in stem swelling.

### Complex regulation of DELLA genes in stem swelling

Light and hormone signals are involved in plant growth and development (Kepinski, 2006; Kami et al., 2010). DELLA proteins are regarded as the key regulators of gibberellin and light signal transduction pathways (Fukazawa et al., 2014; Achard et al., 2003; Feng et al., 2008). Therefore, it is important to explore the regulatory mechanism of DELLA genes in stem swelling in stem mustard. The interaction network of GRAS proteins with other proteins in the genome was constructed to identify their interacting proteins. Eight genes encoding GID1, SLY1, and PIF3 proteins were found to interact with several DELLA genes. It was previously reported that DELLA proteins inhibit plant growth and development by repressing the GA signaling pathways (Park et al., 2013). A study on *SLR1* gene, which encodes a DELLA protein, showed that overexpression of *SLR1* lacking the DELLA domain conferred altered GA responses in rice (Itoh et al., 2005). GID1 family proteins function as GA receptors and play key roles in the degradation of DELLA protein (Ueguchi-Tanaka et al., 2005; Conti et al., 2014). The GID1 genes are divided into particular subfamilies (GID1a, b, c) based on their structural features and evolution (Gazara et al., 2018). The GA–GID1–DELLA interaction is required for GA signaling. Functional characterization showed that GID1a and GID1c retained the canonical GA signaling roles, whereas GID1b interaction with DELLA protein requires less bioactive GA (Murase et al., 2008; Suzuki et al., 2009; Gazara et al., 2018). SLY1 encodes a putative F-box subunit of SCF-type E3 ligase, which recognizes and regulates the proteasomal degradation of DELLAs in GA–GID1–DELLA complex (McGinnis et al., 2003). In our study, two SLY1 proteins (BjuA004160, BjuB014220), and four GID1 proteins, BjuB037910 (belonging to GID1a subfamily), BjuO005843 (belonging to GID1c subfamily), BjuB006276 and BjuB022926 (both belonging to GID1b subfamily), interacted with DELLA proteins in the *B. juncea* genome. The expression of the interacting genes was analyzed across the stages of stem swelling. Compared to the low expression of DELLA genes, the transcripts of GID1 and SLY1 genes were relatively highly expressed, suggesting that the transcriptional regulators DELLA genes may negatively regulate GID1 and SLY1 genes. qRT-PCR also showed that two GID1 genes (*BjuB006276* and *BjuB037910*) were gradually increased during stem swelling process and remained elevated in the later stages. Therefore, it can be deduced that the conserved GID1 genes regulate the DELLA genes by inhibiting their translation during stem swelling in stem mustard.

PIF3 is a key transcription factor that positively regulates the phytochrome signaling pathway (Kim et al., 2003). In *Arabidopsis*, a PIF3-like 5 gene regulates gibberellin responsiveness by directly binding to the promoters of DELLA genes through G-box elements (Oh et al., 2007). In this study, *cis*-elements were identified in the promoter regions of DELLA genes. Almost all DELLA genes contained light-responsive elements such as G-box, I-box, GT1, and Box1. These elements are well-characterized in the regulation of light-mediated development (Zentella et al., 2007). The hormonal regulation elements P-box, GRAE, CGTCA-motif were also identified, providing compelling evidence that DELLA genes interact with other genes to modulate light and hormone signal transduction during plant development.

## Conclusions

In this study, a total of 88 GRAS family members were identified in the *B. juncea* genome and classified into nine groups. Chromosome distribution, gene structures, and motif compositions suggested a complex evolutionary history of this family in *B. juncea*. Functional divergence analysis indicated that GRAS gene family had undergone a selective pressure in the evolutionary rates and amino acid properties. An interaction network of GRAS factors with other genome proteins was constructed to explore the protein interactions. Moreover, the expression patterns of GRAS genes and interactive genes in different development stages during stem swelling were investigated. This study provides new insights into the molecular evolution and biological functions of the GRAS gene family.

##  Supplemental Information

10.7717/peerj.6682/supp-1 Table S1 GRAS genes used in * Arabidopsis*

10.7717/peerj.6682/supp-2 Table S2 Sequences of primers used in qRT-PCR

10.7717/peerj.6682/supp-3 Table S3 Characteristic features of BjuGRAS transcription factors 1: Percentage of Positive amino acid /%; 2:Percentage of Negative amino acid /%; 3: Percentage of Aliphatic amino acid /%; 4: Percentage of Aromatics amino acid /%; GRAVY: Grand average of hydropathicity.

10.7717/peerj.6682/supp-4 Table S4 Chromosomal locations and sequence identify of homologous BjuGRAS genes A: A subgenome; B: B subgenome; U: unanchored contig or scaffold.

10.7717/peerj.6682/supp-5 Table S5 Blast2GO annotation details of BjuGRAS genes

## Additional Information and Declarations

### Data Availability

The following information was supplied regarding data availability: The data is available at NCBI: accession number SRP151320.
