# Supplementary material for: Identification of the GRAS gene family in the Brassica juncea genome provides insight into its role in stem swelling in stem mustard
Source: PeerJ. 2019 Apr 1;7:e6682. doi: 10.7717/peerj.6682 (PMC6448559; doi:10.7717/peerj.6682)
Supplement: Table S1 [file peerj-07-6682-s001.docx]

**Table S1:**

**GRAS genes used in *Arabidopsis*.**

| Gene ID | Name |
| --- | --- |
| AT1G07520 | AtGRAS01 |
| AT1G07530 | AtGRAS02 |
| AT1G14920 | AtGRAS03 |
| AT1G21450 | AtGRAS04 |
| AT1G50420 | AtGRAS05 |
| AT1G50600 | AtGRAS06 |
| AT1G55580 | AtGRAS07 |
| AT1G63100 | AtGRAS08 |
| AT1G66350 | AtGRAS09 |
| AT2G01570 | AtGRAS10 |
| AT2G04890 | AtGRAS11 |
| AT2G29060 | AtGRAS12 |
| AT2G29065 | AtGRAS33 |
| AT2G37650 | AtGRAS13 |
| AT2G45160 | AtGRAS14 |
| AT3G03450 | AtGRAS15 |
| AT3G13840 | AtGRAS16 |
| AT3G46600 | AtGRAS17 |
| AT3G49950 | AtGRAS18 |
| AT3G50650 | AtGRAS19 |
| AT3G54220 | AtGRAS20 |
| AT3G60630 | AtGRAS21 |
| AT4G00150 | AtGRAS22 |
| AT4G08250 | AtGRAS23 |
| AT4G17230 | AtGRAS24 |
| AT4G36710 | AtGRAS25 |
| AT4G37650 | AtGRAS26 |
| AT5G17490 | AtGRAS27 |
| AT5G41920 | AtGRAS28 |
| AT5G48150 | AtGRAS29 |
| AT5G52510 | AtGRAS30 |
| AT5G59450 | AtGRAS31 |
| AT5G66770 | AtGRAS32 |
