# Supplementary material for: Identification of the GRAS gene family in the Brassica juncea genome provides insight into its role in stem swelling in stem mustard
Source: PeerJ. 2019 Apr 1;7:e6682. doi: 10.7717/peerj.6682 (PMC6448559; doi:10.7717/peerj.6682)
Supplement: Table S2 [file peerj-07-6682-s002.docx]

**Table S2:**

**Sequences of primers used in qRT-PCR.**

| **Gene ID** | **Forward primer (5'→3')** | **Reverse primer (5'→3')** |
| --- | --- | --- |
| *BjuGRAS3* | CTCCGCCGCAGACTAAGAT | TTAGCCACGAAGCCACGATA |
| *BjuGRAS5* | AAGGAAGAAGAAGACGGTGGAA | CGACGAGGAGGAAGAATCAATC |
| *BjuGRAS7* | GCGTATAAGCAAGCGAGTATGT | GCAGATGTAGCGATTAGTGGTC |
| *BjuGRAS8* | GCAACCTAACACCATTCTCAGA | GTCAACGCCGAGCATTACC |
| *BjuGRAS10* | TTGCTCGCCTCGTCACAA | AACCGCTATGGATACAGAACCT |
| *BjuB006276* | CACCAACCTCCTCACCAGAA | AACCATCGTCGTAAGCACAAG |
| *BjuB037910* | TGAGGCACTGGCGTTGTT | CTCTCGGCGTAGGCTTGAA |
| *BjuA004160* | ACTCTACGAGGTGTTGAAGCA | CGCCAAGACGACGAATCTG |
| *BjuA021658* | AGAAGAAGCAATGGCGATGAAT | ACGGTTCCTGTCTCTGAGTG |
| *Actin2* | ATCGTCTGTGACAACGGTAC | GATACGGAGCTCGTTGTAGA |
