# Supplementary material for: Identification of the GRAS gene family in the Brassica juncea genome provides insight into its role in stem swelling in stem mustard
Source: PeerJ. 2019 Apr 1;7:e6682. doi: 10.7717/peerj.6682 (PMC6448559; doi:10.7717/peerj.6682)
Supplement: Table S3 — 1: Percentage of Positive amino acid /%; 2:Percentage of Negative amino acid /%; 3: Percentage of Aliphatic amino acid /%; 4: Percentage of Aromatics amino acid /%; GRAVY: Grand average of hydropathicity. [file peerj-07-6682-s003.docx]

**Table S3:**

**Characteristic features of BjuGRAS transcription factors.**

1: Percentage of Positive amino acid /%; 2:Percentage of Negative amino acid /%; 3: Percentage of Aliphatic amino acid /%; 4: Percentage of Aromatics amino acid /%; GRAVY: Grand average of hydropathicity.

| Group | Name | Gene ID | Size (aa) | Mw (KDa) | pI | I,L,V^1^ | F,W,Y^2^ | K,R,H^3^ | D,E^4^ | GRAVY |
| --- | --- | --- | --- | --- | --- | --- | --- | --- | --- | --- |
| DELLA | BjuGRAS1 | BjuA021102 | 544 | 59.33 | 4.72 | 22 | 7 | 10 | 14 | -0.222 |
|  | BjuGRAS2 | BjuA046262 | 575 | 62.80 | 5.46 | 21 | 7 | 11 | 11 | -0.181 |
|  | BjuGRAS3 | BjuA033945 | 579 | 63.31 | 5.33 | 20 | 7 | 11 | 12 | -0.235 |
|  | BjuGRAS4 | BjuA024803 | 571 | 62.30 | 5.66 | 20 | 8 | 12 | 11 | -0.19 |
|  | BjuGRAS5 | BjuB027815 | 587 | 64.10 | 5.37 | 20 | 7 | 11 | 12 | -0.235 |
|  | BjuGRAS6 | BjuA046122 | 530 | 57.84 | 4.81 | 21 | 7 | 10 | 13 | -0.241 |
|  | BjuGRAS7 | BjuO004850 | 508 | 56.19 | 5.37 | 24 | 8 | 12 | 13 | -0.165 |
|  | BjuGRAS8 | BjuA047224 | 510 | 55.85 | 4.74 | 23 | 7 | 10 | 14 | -0.154 |
|  | BjuGRAS9 | BjuB035481 | 512 | 56.03 | 4.81 | 23 | 7 | 10 | 13 | -0.105 |
|  | BjuGRAS10 | BjuA007186 | 507 | 55.97 | 5.31 | 24 | 8 | 12 | 12 | -0.121 |
|  | Average |  | 542 | 59.37 | 5.16 | 22 | 7 | 11 | 13 | -0.185 |
| HAM | BjuGRAS11 | BjuA040758 | 489 | 54.46 | 5.04 | 22 | 9 | 11 | 13 | -0.145 |
|  | BjuGRAS12 | BjuB040790 | 484 | 53.91 | 5.05 | 22 | 9 | 11 | 13 | -0.132 |
|  | BjuGRAS13 | BjuA038356 | 487 | 54.11 | 5.58 | 22 | 8 | 12 | 11 | -0.188 |
|  | BjuGRAS14 | BjuB047815 | 481 | 53.30 | 5.52 | 21 | 8 | 12 | 12 | -0.214 |
|  | BjuGRAS15 | BjuB022360 | 606 | 66.92 | 5.34 | 21 | 8 | 10 | 10 | -0.318 |
|  | BjuGRAS16 | BjuB019362 | 627 | 69.22 | 5.59 | 23 | 7 | 11 | 10 | -0.204 |
|  | BjuGRAS17 | BjuO012628 | 490 | 54.23 | 5.58 | 23 | 9 | 12 | 12 | -0.086 |
|  | BjuGRAS18 | BjuA026646 | 602 | 66.44 | 5.26 | 20 | 9 | 10 | 10 | -0.322 |
|  | BjuGRAS19 | BjuA031378 | 562 | 61.77 | 5.79 | 23 | 7 | 11 | 9 | -0.138 |
|  | BjuGRAS20 | BjuB016848 | 605 | 66.67 | 5.26 | 21 | 9 | 10 | 11 | -0.239 |
|  | BjuGRAS21 | BjuA010717 | 605 | 66.59 | 5.36 | 21 | 9 | 10 | 11 | -0.232 |
|  | BjuGRAS22 | BjuA014518 | 550 | 60.30 | 4.71 | 19 | 9 | 8 | 11 | -0.272 |
|  | BjuGRAS23 | BjuB023017 | 580 | 63.43 | 4.81 | 20 | 8 | 9 | 11 | -0.298 |
|  | Average |  | 551 | 60.87 | 5.30 | 21 | 8 | 11 | 11 | -0.214 |
| LISCL | BjuGRAS24 | BjuB008137 | 594 | 67.35 | 6.08 | 19 | 9 | 15 | 14 | -0.477 |
|  | BjuGRAS25 | BjuA038604 | 599 | 67.59 | 6.03 | 19 | 9 | 14 | 14 | -0.448 |
|  | BjuGRAS26 | BjuB016391 | 667 | 75.04 | 6.39 | 20 | 9 | 16 | 14 | -0.442 |
|  | BjuGRAS27 | BjuB019585 | 558 | 63.60 | 5.61 | 18 | 9 | 14 | 14 | -0.45 |
|  | BjuGRAS28 | BjuA042028 | 673 | 75.56 | 6.33 | 20 | 9 | 15 | 14 | -0.447 |
|  | BjuGRAS29 | BjuA031260 | 760 | 85.60 | 5.53 | 17 | 11 | 13 | 13 | -0.504 |
|  | BjuGRAS30 | BjuB033474 | 792 | 88.74 | 5.53 | 18 | 10 | 13 | 13 | -0.52 |
|  | BjuGRAS31 | BjuO013267 | 707 | 80.28 | 6.53 | 18 | 11 | 14 | 13 | -0.515 |
|  | BjuGRAS32 | BjuA026353 | 705 | 79.97 | 6.10 | 18 | 10 | 15 | 13 | -0.545 |
|  | BjuGRAS33 | BjuA016395 | 722 | 82.21 | 6.35 | 19 | 11 | 14 | 13 | -0.536 |
|  | BjuGRAS34 | BjuA019478 | 666 | 76.47 | 5.75 | 18 | 11 | 13 | 13 | -0.55 |
|  | BjuGRAS35 | BjuA026354 | 619 | 71.54 | 6.22 | 20 | 10 | 16 | 15 | -0.515 |
|  | BjuGRAS36 | BjuO013266 | 633 | 72.83 | 5.35 | 21 | 9 | 15 | 16 | -0.461 |
|  | BjuGRAS37 | BjuB015393 | 667 | 76.68 | 5.61 | 18 | 11 | 13 | 14 | -0.544 |
|  | BjuGRAS38 | BjuA019477 | 690 | 78.63 | 5.79 | 19 | 10 | 14 | 14 | -0.559 |
|  | BjuGRAS39 | BjuB015391 | 667 | 76.75 | 5.55 | 18 | 11 | 13 | 14 | -0.548 |
|  | BjuGRAS40 | BjuB015394 | 698 | 79.82 | 6.08 | 18 | 10 | 15 | 14 | -0.583 |
|  | BjuGRAS41 | BjuO006829 | 444 | 50.71 | 5.34 | 21 | 9 | 12 | 13 | -0.346 |
|  | Average |  | 659 | 74.97 | 5.90 | 19 | 10 | 14 | 14 | -0.499 |
| LS | BjuGRAS42 | BjuA046167 | 442 | 49.17 | 6.17 | 21 | 9 | 12 | 10 | -0.147 |
|  | BjuGRAS43 | BjuO003237 | 444 | 49.52 | 6.43 | 21 | 9 | 12 | 10 | -0.186 |
|  | BjuGRAS44 | BjuB020878 | 600 | 65.65 | 4.66 | 21 | 8 | 9 | 13 | -0.34 |
|  | BjuGRAS45 | BjuO005616 | 596 | 64.77 | 4.73 | 21 | 9 | 9 | 12 | -0.259 |
|  | BjuGRAS46 | BjuB018204 | 525 | 58.77 | 5.35 | 22 | 9 | 11 | 12 | -0.292 |
|  | Average |  | 521 | 57.58 | 5.47 | 21 | 9 | 11 | 11 | -0.245 |
| PAT1 | BjuGRAS47 | BjuB042895 | 497 | 55.75 | 6.34 | 20 | 8 | 14 | 12 | -0.345 |
|  | BjuGRAS48 | BjuA008320 | 497 | 55.85 | 6.30 | 20 | 8 | 14 | 12 | -0.355 |
|  | BjuGRAS49 | BjuB044594 | 498 | 55.77 | 6.00 | 20 | 9 | 13 | 12 | -0.285 |
|  | BjuGRAS50 | BjuO012383 | 523 | 58.16 | 5.56 | 20 | 8 | 12 | 12 | -0.361 |
|  | BjuGRAS51 | BjuA021405 | 525 | 58.37 | 5.71 | 20 | 7 | 12 | 12 | -0.404 |
|  | BjuGRAS52 | BjuB047031 | 527 | 58.72 | 5.64 | 20 | 8 | 12 | 12 | -0.383 |
|  | BjuGRAS53 | BjuB031392 | 565 | 63.02 | 5.04 | 20 | 8 | 12 | 14 | -0.396 |
|  | BjuGRAS54 | BjuA043110 | 577 | 64.51 | 4.89 | 19 | 8 | 12 | 15 | -0.399 |
|  | BjuGRAS55 | BjuB017943 | 578 | 64.33 | 4.93 | 19 | 8 | 11 | 14 | -0.376 |
|  | BjuGRAS56 | BjuA026060 | 541 | 60.17 | 5.13 | 21 | 8 | 12 | 14 | -0.322 |
|  | BjuGRAS57 | BjuA019263 | 346 | 38.86 | 6.91 | 24 | 8 | 15 | 12 | -0.209 |
|  | BjuGRAS58 | BjuA004915 | 530 | 58.84 | 5.90 | 20 | 9 | 13 | 12 | -0.266 |
|  | BjuGRAS59 | BjuB013253 | 542 | 59.82 | 6.09 | 19 | 8 | 13 | 11 | -0.315 |
|  | BjuGRAS60 | BjuB009129 | 528 | 58.40 | 5.68 | 19 | 9 | 13 | 12 | -0.28 |
|  | BjuGRAS61 | BjuA000601 | 530 | 58.47 | 5.53 | 19 | 9 | 12 | 12 | -0.261 |
|  | BjuGRAS62 | BjuA025918 | 318 | 35.79 | 5.90 | 24 | 9 | 14 | 13 | -0.097 |
|  | BjuGRAS63 | BjuB007739 | 453 | 50.79 | 5.61 | 19 | 8 | 12 | 12 | -0.352 |
|  | BjuGRAS64 | BjuA038107 | 608 | 65.47 | 5.56 | 21 | 6 | 11 | 11 | -0.308 |
|  | BjuGRAS65 | BjuB035207 | 617 | 67.22 | 5.92 | 21 | 6 | 11 | 11 | -0.298 |
|  | BjuGRAS66 | BjuA006997 | 576 | 62.16 | 5.57 | 21 | 5 | 12 | 12 | -0.269 |
|  | Average |  | 519 | 57.52 | 5.71 | 20 | 8 | 13 | 12 | -0.314 |
| SCL28 | BjuGRAS67 | BjuA046833 | 651 | 73.15 | 7.33 | 19 | 8 | 15 | 12 | -0.536 |
|  | BjuGRAS68 | BjuB028332 | 659 | 73.40 | 6.81 | 19 | 7 | 15 | 12 | -0.533 |
|  | BjuGRAS69 | BjuA044545 | 486 | 53.92 | 8.45 | 17 | 7 | 16 | 11 | -0.57 |
|  | Average |  | 599 | 66.82 | 7.53 | 18 | 7 | 15 | 12 | -0.546 |
| SCL3 | BjuGRAS70 | BjuA028017 | 445 | 49.74 | 6.03 | 23 | 7 | 13 | 11 | -0.203 |
|  | BjuGRAS71 | BjuO012393 | 442 | 49.56 | 5.84 | 23 | 8 | 13 | 12 | -0.177 |
|  | BjuGRAS72 | BjuB034966 | 444 | 49.79 | 6.03 | 23 | 7 | 13 | 12 | -0.207 |
|  | BjuGRAS73 | BjuB047044 | 444 | 49.79 | 6.25 | 23 | 8 | 14 | 12 | -0.237 |
|  | BjuGRAS74 | BjuA021416 | 444 | 49.99 | 6.05 | 23 | 8 | 14 | 12 | -0.207 |
|  | Average |  | 444 | 49.77 | 6.05 | 23 | 8 | 13 | 12 | -0.206 |
| SCR | BjuGRAS75 | BjuB046263 | 640 | 70.11 | 5.83 | 20 | 6 | 11 | 10 | -0.358 |
|  | BjuGRAS76 | BjuA035682 | 626 | 68.38 | 5.68 | 20 | 6 | 11 | 10 | -0.355 |
|  | BjuGRAS77 | BjuA015784 | 404 | 44.46 | 5.47 | 24 | 9 | 11 | 12 | -0.071 |
|  | BjuGRAS78 | BjuB025820 | 408 | 44.90 | 5.42 | 25 | 8 | 12 | 12 | -0.089 |
|  | Average |  | 520 | 56.96 | 5.60 | 22 | 7 | 11 | 11 | -0.218 |
| SHR | BjuGRAS79 | BjuB038552 | 531 | 59.65 | 5.57 | 17 | 10 | 13 | 13 | -0.425 |
|  | BjuGRAS80 | BjuA014265 | 532 | 59.83 | 5.58 | 17 | 10 | 13 | 13 | -0.444 |
|  | BjuGRAS81 | BjuB041976 | 530 | 59.42 | 5.38 | 17 | 9 | 13 | 13 | -0.381 |
|  | BjuGRAS82 | BjuA002831 | 533 | 59.76 | 5.61 | 17 | 9 | 13 | 12 | -0.43 |
|  | BjuGRAS83 | BjuB040830 | 530 | 59.36 | 5.45 | 17 | 9 | 12 | 13 | -0.422 |
|  | BjuGRAS84 | BjuA043760 | 525 | 58.97 | 5.54 | 17 | 9 | 13 | 13 | -0.422 |
|  | BjuGRAS85 | BjuB006058 | 415 | 46.68 | 5.80 | 24 | 9 | 11 | 11 | -0.022 |
|  | BjuGRAS86 | BjuA044464 | 373 | 41.94 | 5.95 | 24 | 9 | 11 | 10 | -0.053 |
|  | BjuGRAS87 | BjuB023885 | 511 | 57.36 | 5.21 | 20 | 9 | 13 | 14 | -0.443 |
|  | BjuGRAS88 | BjuA038913 | 509 | 57.10 | 4.89 | 20 | 9 | 12 | 15 | -0.423 |
|  | Average |  | 499 | 56.01 | 5.50 | 19 | 9 | 12 | 13 | -0.347 |
|  | Total |  | 551 | 61.48 | 5.65 | 23 | 8 | 12 | 12 | -0.320 |
