# Supplementary material for: Identification of the GRAS gene family in the Brassica juncea genome provides insight into its role in stem swelling in stem mustard
Source: PeerJ. 2019 Apr 1;7:e6682. doi: 10.7717/peerj.6682 (PMC6448559; doi:10.7717/peerj.6682)
Supplement: Table S4 — A: A subgenome; B: B subgenome; U: unanchored contig or scaffold. [file peerj-07-6682-s004.docx]

**Table S4:**

**Chromosomal locations and sequence identify of homologous BjuGRAS genes.**

A: A subgenome; B: B subgenome; U: unanchored contig or scaffold.

| Number | Group | Gene 1 | Location | Gene 2 | Location | Sequence Identity (%) |
| --- | --- | --- | --- | --- | --- | --- |
| 1 | DELLA | BjuGRAS1 | A | BjuGRAS6 | A | 92 |
| 2 | DELLA | BjuGRAS8 | A | BjuGRAS9 | B | 92 |
| 3 | DELLA | BjuGRAS10 | A | BjuGRAS7 | U | 95 |
| 4 | DELLA | BjuGRAS3 | A | BjuGRAS5 | B | 94 |
| 5 | DELLA | BjuGRAS2 | A | BjuGRAS4 | A | 91 |
| 6 | SCL3 | BjuGRAS70 | A | BjuGRAS72 | B | 96 |
| 7 | SCL3 | BjuGRAS74 | A | BjuGRAS73 | B | 93 |
| 8 | LS | BjuGRAS42 | A | BjuGRAS43 | U | 95 |
| 9 | LS | BjuGRAS44 | B | BjuGRAS45 | U | 91 |
| 10 | SCL28 | BjuGRAS69 | A | BjuGRAS68 | B | 91 |
| 11 | PAT1 | BjuGRAS64 | A | BjuGRAS65 | B | 92 |
| 12 | PAT1 | BjuGRAS61 | A | BjuGRAS60 | B | 91 |
| 13 | PAT1 | BjuGRAS58 | A | BjuGRAS59 | B | 91 |
| 14 | PAT1 | BjuGRAS48 | A | BjuGRAS47 | B | 96 |
| 15 | PAT1 | BjuGRAS51 | A | BjuGRAS52 | B | 96 |
| 16 | PAT1 | BjuGRAS54 | A | BjuGRAS55 | B | 90 |
| 17 | SCR | BjuGRAS76 | A | BjuGRAS75 | B | 90 |
| 18 | SCR | BjuGRAS77 | A | BjuGRAS78 | B | 90 |
| 19 | SHR | BjuGRAS80 | A | BjuGRAS79 | B | 98 |
| 20 | SHR | BjuGRAS82 | A | BjuGRAS83 | B | 97 |
| 21 | SHR | BjuGRAS84 | A | BjuGRAS81 | B | 91 |
| 22 | SHR | BjuGRAS86 | A | BjuGRAS85 | B | 93 |
| 23 | SHR | BjuGRAS88 | A | BjuGRAS87 | B | 92 |
| 24 | HAM | BjuGRAS11 | A | BjuGRAS12 | B | 92 |
| 25 | HAM | BjuGRAS13 | A | BjuGRAS14 | B | 91 |
| 26 | HAM | BjuGRAS18 | A | BjuGRAS15 | B | 93 |
| 27 | HAM | BjuGRAS16 | A | BjuGRAS19 | B | 88 |
| 28 | HAM | BjuGRAS21 | A | BjuGRAS20 | B | 98 |
| 29 | HAM | BjuGRAS22 | A | BjuGRAS23 | B | 88 |
| 30 | HAM | BjuGRAS25 | A | BjuGRAS24 | B | 91 |
| 31 | LISCL | BjuGRAS28 | A | BjuGRAS26 | B | 91 |
| 32 | LISCL | BjuGRAS29 | A | BjuGRAS30 | B | 90 |
| 33 | LISCL | BjuGRAS32 | A | BjuGRAS31 | U | 91 |
| 34 | LISCL | BjuGRAS34 | A | BjuGRAS37 | B | 91 |
| 35 | LISCL | BjuGRAS34 | A | BjuGRAS39 | B | 91 |
| 36 | LISCL | BjuGRAS37 | B | BjuGRAS39 | B | 100 |
| 37 | LISCL | BjuGRAS38 | A | BjuGRAS40 | B | 97 |
| 38 | LISCL | BjuGRAS35 | A | BjuGRAS36 | U | 92 |
