# Supplementary material for: Identification of the GRAS gene family in the Brassica juncea genome provides insight into its role in stem swelling in stem mustard
Source: PeerJ. 2019 Apr 1;7:e6682. doi: 10.7717/peerj.6682 (PMC6448559; doi:10.7717/peerj.6682)
Supplement: Table S5 [file peerj-07-6682-s005.docx]

**Table S5:**

**Blast2GO annotation details of BjuGRAS genes.**

| Number of annotations per gene | Number of genes | Gene name |
| --- | --- | --- |
| 0 | 29 | BjuGRAS85, 24, 47, 48, 26, 27, 20, 86, 21, 28, 29, 30, 35, 58, 36, 14, 37, 38, 31, 53, 32, 54, 33, 77, 78, 34, 39, 17, 19 |
| 1 | 0 |  |
| 2 | 5 | BjuGRAS71, 63, 13, 49, 2 |
| 3 | 2 | BjuGRAS87, 88 |
| 4 | 3 | BjuGRAS72, 73, 70 |
| 5 | 11 | BjuGRAS62, 40, 41, 46, 25, 42, 43, 55, 44, 56, 45 |
| 6 | 16 | BjuGRAS60, 83, 61, 50, 51, 74, 52, 79, 68, 57, 69, 59, 64, 65, 66, 67 |
| 7 | 2 | BjuGRAS11, 12 |
| 8 | 2 | BjuGRAS75, 76 |
| 9 | 0 |  |
| 10 | 9 | BjuGRAS82, 84, 18, 80, 81, 15, 16, 22, 23 |
| 11 | 4 | BjuGRAS1, 7, 6, 10 |
| 12 | 2 | BjuGRAS9, 8 |
| 13 | 0 |  |
| 14 | 0 |  |
| 15 | 3 | BjuGRAS3, 4, 5 |
